# Exploring elderly patients’ experiences and concerns about early mobilization implemented in postoperative care following lumbar spinal surgery: a qualitative study

**DOI:** 10.1186/s12912-023-01510-7

**Published:** 2023-10-04

**Authors:** Jie Huang, Pan Li, Huiting Wang, Chenxi Lv, Jing Han, Xuemei Lu

**Affiliations:** 1 Spine Department, Beijing Jishuitan Hospital, Capital Medical University, No. 31, Xinjiekou East Street, Xicheng District, Beijing, China; 2 School of Nursing, Xuzhou Medical University, Jiangsu Province, China; 3 Nursing Department, Beijing Jishuitan Hospital, Capital Medical University, Beijing, China

**Keywords:** Early mobilization, Elderly, Lumbar, Education, Pain management

## Abstract

**Background:**

Given its apparent benefits, early mobilization is becoming increasingly important in spinal surgery. However, the time point at which patients first get out of bed for mobilization after spinal surgery varies widely. Beginning in January 2022, we conducted a study of early mobilization (mobilization within 4 h postoperatively) following multi-segment lumbar decompression and fusion surgery in elderly patients. The study goal was to better understand elderly patients’ perceptions of early mobilization and ultimately contribute to the improvement of elderly patients’ perioperative experiences and quality of life.

**Methods:**

We employed a qualitative descriptive study design involving face-to-face semi-structured interviews. Forty-five consecutive patients were invited, among whom 24 were enrolled and completed the qualitative investigation from February to June 2022. Of these 24 patients, 10 underwent early mobilization (mobilization within 4 h postoperatively) and 14 underwent mobilization at ≥ 24 h postoperatively. Three researchers conducted a 15-question interview the day before each patient’s discharge. The interviews were audio-recorded, and content analysis was used to assess the data.

**Results:**

Six themes regarding the patients’ experiences and concerns about early mobilization were identified: worries, benefits, daily routines, pain, education, and support. The study results revealed the obstacles in early mobilization practice and highlighted the importance of perioperative education on early mobilization.

**Conclusions:**

Clear and explicit guidance on early mobilization and a multidisciplinary mobilization protocol that incorporates a comprehensive pain management plan are essential for effective patient education. These measures may have positive effects on reducing patients’ stress and anxiety regarding postoperative early mobilization.

## Background

The concept of early mobilization is complex and interdisciplinary, consisting of both physical and psychological aspects [1]. It is generally defined as a technique in which the patient gets out of bed as soon as possible after surgery. In recent years, early mobilization has been strongly recommended as part of enhanced recovery after orthopaedic surgery [2]. The clinical practice guideline issued by the American Association of Neuroscience Nurses recommends mobilization of orthopaedic patients soon after spinal surgery unless complications arise or unless otherwise specified [3].

Immobilization or bed rest after orthopaedic surgical procedures has been demonstrated to be accompanied by problems such as urinary tract infection, pneumonia, and venous thromboembolism, which are potentially lethal complications [4, 5]. Early mobilization is safe and feasible, and it has positive results that lead to a shorter length of stay, lower healthcare costs, and a lower complication rate [6, 7]. Furthermore, early mobilization can not only enhance patient experiences by reducing pain but also improve performance-based function and patient satisfaction [8–10].

Early mobilization protocols have been successfully established and applied, and many have shown promising results. A study involving 406 consecutive patients following total knee arthroplasty showed that early mobilization could help reduce the length of stay, decrease postoperative complications, and improve functional outcomes [11]. Another study involving 240 patients undergoing hip fracture surgery showed that early mobilization was as safe as delayed mobilization in terms of the complication rate and indicated that early mobilization could help to reduce the postoperative length of stay and financial costs [12]. Additionally, in a pilot study conducted in a surgical/trauma intensive care unit in the southeast United States, the researchers applied their mobility protocol involving six activity events for mechanically ventilated patients. The patients maintained stable vital signs, no extubation or line removal events occurred, and the length of stay was shorter than average [13].

Given the apparent benefits of early mobilization in orthopaedic surgery, it is not surprising that implementation of early mobilization in spinal surgery is becoming increasingly common. Decompression and fusion surgery under general anaesthesia is an effective treatment for degenerative, traumatic, and oncological pathologies of the spine [14]. In a randomized controlled study by Qvarfordh et al. [15], early mobilization after lumbar discectomy benefited patients by decreasing the need for analgesia and oxygen supplementation on the first postoperative day. In a study by Adogwa et al. [6] involving 125 elderly patients undergoing surgery for correction of adult degenerative scoliosis of the spine, early mobilization helped decrease the length of hospital stay, decrease perioperative complications, and improve functional outcomes. Our study team found that early mobilization in elderly patients undergoing lumbar decompression and fusion surgery contributed to an improved postoperative functional status, lower incidence of complications, and shorter postoperative hospital stay [16]. Additionally, the results of a study conducted by Weerink et al. [17] suggested that early mobilization was a successful and safe alternative to immobilization in nonoperative treatment of elderly patients with spinal fractures involving both the middle and anterior columns based on the fact that early mobilization did not lead to new complications or neurological damage.

The concept of early mobilization after spinal surgery is widely accepted, and its benefits have been demonstrated. However, there are still obstacles to the application of early mobilization in actual clinical practice. The time point at which patients first get out of bed for mobilization after spinal surgery varies widely because patients come from different countries and regions, patients exhibit individual diversity in size and other factors, the number of healthcare providers involved in patients’ medical care varies, and no specific recommendations are available regarding how soon patients should get out of bed for mobilization. The time point of early mobilization ranges from a few hours to several days and even weeks. Surgeons’ and nurses’ decisions are also crucial, and they are generally based on surgical techniques and traditions in nursing practice.

We conducted the present study of early mobilization in elderly patients following multi-segment lumbar decompression and fusion surgery beginning in January 2022 (ClinicalTrials.gov ID: NCT04133103). The patients in the early mobilization group began mobilization within 4 h postoperatively assisted by a nurse. They walked around the corridor of the ward and could return to bed at any time to avoid fatigue. We had no requirements regarding the duration or number of times they should mobilize during the first postoperative mobilization. We found that the beneficial results of early mobilization were similar to those in other studies, including studies of spinal and nonspinal surgeries. However, all previous studies were quantitative in nature; no qualitative studies on postoperative early mobilization have yet been performed. Qualitative approaches should be considered to better understand elderly patients’ perceptions of early mobilization after multi-segment lumbar decompression and fusion surgery and ultimately contribute to the improvement of elderly patients’ perioperative experiences and quality of life. Gaining patients’ insights may generate new information on strategies for the clinical practice of early mobilization after spinal surgery. The current research aims to gain a rich and more in-depth understanding of elderly patients’ experiences and concerns regarding early mobilization following multi-segment lumbar decompression and fusion surgery.

## Methods

### Aim and design

This qualitative investigation was based on phenomenological research, using semi-structured interviews focused on elderly patients’ experiences and concerns about early mobilization after multi-segment lumbar decompression and fusion surgery.

### Setting and participants

This study was conducted in the National Center for Orthopaedics in China, which is a > 1,700-bed academic medical centre. This centre has a 150-bed spine unit and is located in central Beijing, China. It is one of the best spine units in China and performs an average of 4,000 spinal surgeries annually. Patients visit this centre from all over China.

Patients undergoing lumbar decompression and fusion surgery at the spine unit were invited to participate in the current qualitative investigation. The inclusion criteria were willingness to join this study, ability to communicate in Mandarin, age of ≥ 60 years, and treatment by multi-segment lumbar decompression and fusion surgery. The exclusion criteria were spinal cord injury, cognitive impairment, and mental illness. Forty-five consecutive patients were invited, and purposive sampling was performed. Twenty-four patients expressed their interest in participating and completed the interview until data saturation was reached, signifying that no new themes emerged. This study was approved by the ethics committee of our hospital (No. 202202-06) and strictly adhered to the tenets of the Declaration of Helsinki. All participating patients received an information sheet describing the purpose of this current study and the benefits to the participant. All participating patients provided written informed consent.

### Data collection

Patients were screened and interviewed by three female researchers aged 34 to 35 years. All three researchers had > 10 years of clinical nursing experience and > 4 years of scientific research training in our hospital. The interviewers had no relationship with the participants, and the participants were not compensated for participation in this study. The interviewers attended the participants’ daily morning rounds with the doctors in charge and thus developed a relationship of trust with the participants. The day before discharge, an in-depth semi-structured interview was conducted face-to-face in a private conversation room at a time chosen to accommodate the participants’ comfort.

The interview questions were predetermined by a group of psychologists, anaesthesiologists, nurses, and surgeons (Table 1). An interview guide was used to ensure that the areas of prior interest were explored in each interview. After the interview, the study researchers summarized the crucial points of the interview with the participants to ensure accuracy and solicit further comments. The interviews were audio-recorded for subsequent transcription, lasted between 30 and 60 min, and were completed from February to June 2022.

**Table 1:** Semi-structured interview guide

Questions	Follow-up questions
1. Think back to before surgery, what is your expectation for operation?	
2. What challenging or difficult you were facing before surgery?	
3. Think back to before surgery, did the hospital, surgeons, or nurses do anything to help you acquaint with the information about the operation and the postoperative rehabilitations?	3A: When did you get the information? 3B: What information did you obtain from them? 3C: Who accompanied you to get the information? Do you think it is necessary to bring others with you together to get the information? 3D: What did you like/dislike about the presurgical education?
4. What challenging or difficult you were facing after surgery?	Did anything surprise you or go wrong?
5. What is your expectation for postoperative ambulation?	5A: How long after surgery do you think is the appropriate time to get up out of bed? 5B: How long did it take for you to feel wholly recovered (if at all)?
6. Did the hospital, surgeons, or nurses do anything to help you prepare or cope for the first-time postoperative ambulation?	6A: What did they do to help you? 6B: What did you like/dislike about the current preparation?
7. Did the hospital, surgeons, or nurses introduce you to the postoperative early ambulation program?	7A: When did you know about the program? 7B: In what form? (e.g., oral, paper, video, APP) 7C: Could you fully understand the program? 7D: What other information about postoperative ambulation do you want to obtain from surgeons or nurses?
8. Did you take part in the postoperative early ambulation program?	8A: What was the reason for you to take part in nor not take part in the postoperative early ambulation program? 8B: When was the first time for you to get out of bed ambulation? 8C: Did anything surprise you or go wrong in your ambulation? 8D: Was there anything you were not prepared for?
9. We want to know your thoughts, behaviors, and feelings that were challenging or difficult for you regarding postoperative ambulation.	9A: Did any thoughts or worries bother you after surgery? 9B: What emotions were the hardest or most uncomfortable to deal with? 9C: Did you use any methods to help you feel better?
10. Did you suffer from extra pain during or after ambulation?	Did you stop the next time ambulation?
11. Did you use any strategies to manage the pain you experienced during or after ambulation?	What have you or others done to make the pain better or go away?
12. What did you or your family need during this period to help you with ambulation?	
13. Do you think the postoperative early ambulation program is helpful for you?	13A: What would such a program most concerned do you think? 13B: Is the program gets you benefits?
14. What other information regarded to ambulate you were getting from other channels?	
15. Is there anything else you want to share with us?	

### Data analysis

The study researchers transcribed the audio-recorded interview contents. The data were analysed using Dedoose software, which is a data management program designed for content analysis. In the initial analysis, two transcripts were randomly selected from among the participants to summarize the findings and delineate substantive themes discussed by the participants [18, 19]. This was a process of open coding, creation of categories, and abstraction. The emerging themes were compared and discussed among the researchers to ensure that they achieved similar themes or set a new one. An initial codebook was then developed according to these themes and used to code the remaining interviews. The transcripts were then systematically coded. The initial codebook was modified according to the newly identified themes emerging from the analysis of new interviews and could be revised if the researcher redefined the current codes to include new information or determined distinct concepts. Upon the completion of coding of all interviews, similar or dissimilar themes were ordered into more prominent themes, which were named using content-characteristic words or general thoughts or concepts [18]. The researchers reread, recoded, and reanalysed the interviews, and they then held a discussion each week for four consecutive weeks until the themes exhibited breadth and depth. The number of themes was refined from eight to a final total of six. The Consolidated Criteria for Reporting Qualitative Research (COREQ) was used to improve the validity and rigour of the report [20]. The audio records, transcriptions, and analysis documents were securely stored.

The quotes presented in this paper were translated into English and slightly adapted to maintain meaning by one researcher and then re-translated by another researcher. Both researchers were fluent in Mandarin and English.

## Results

Twenty-four patients were enrolled and completed this study (Table 2). Six themes were finally identified: worries, benefits, daily routines, pain, education, and support (Table 3).

**Table 2:** Participant demographics (N = 24)

Variable	Median(Interquartile range) or No.(%)
Age, y	66.5 (5.3)
Gender	
Male	9 (37.5)
Female	15 (62.5)
Habitats	
Suburban	15 (62.5)
Downtown	9 (37.5)
Marital status	
Married	21 (87.5)
Single	1(4.2)
Divorced	2 (8.3)
Education	
Illiterate	4 (16.6)
High school or below	10 (41.7)
College or below	7 (29.2)
Bachelor or above	3 (12.5)
Clinical diagnosis Lumbar Spinal Stenosis	18 (75.0)
Lumbar Disc Herniation	6 (25.0)
Pain score before surgery	3 (4)
American Society of Anesthesiologists (ASA)	2 (0)
Length of stay after surgery, d	4.0 (1.0)

**Table 3:** Themes and subthemes

Themes	Subthemes
1. Worries	1A. Fear, stress, insecurity
	1B. Screw failure
	1C. Bone recovery
	1D. Monitoring
2. Benefits	2A. Rapid recovery
	2B. Physical functions
3. Daily routines	3A. Sleeping posture
	3B. Get up
	3C. Exercise
	3D. Diet
4. Pain	4A. Postoperative pain
	4B. Pain management strategies
	4C. Medications
5. Education	5A. Patient education
	5B. Inconsistency
	5C. Trust for online information
	5D. Trust for other’s experiences
6. Supports	6A. Family supports
	6B. Social supports

### Worries

Understandably, many patients feel anxious about the adverse effects that could result from mobilization early after spinal surgery. Almost all participants interviewed in this study were worried about the impact of early mobilization on their recovery process after lumbar decompression and fusion surgery, as well as whether this procedure would result in adverse physical outcomes such as intolerable pain, numbness, and paralysis. These worries affected their enthusiasm for early mobilization.

*“I was very nervous about this thing (early mobilization) in my mind. My daughter told me that I must listen to what the doctors and nurses told me. Nevertheless, you know, I tend to be an anxious person, and I could not stop my anxiety.” – Interviewee 5, female, 62 years old*.

*“Maybe it (early mobilization) is work for others, but I do not believe this. Can you ensure my safety? I have just had a major surgery, you know.” – Interviewee 2, male, 68 years old*.

Regardless of their worries and concerns, the participants also shared their positive opinions about early mobilization. They believed that early mobilization would result in a relatively rapid recovery process.

*“Early mobilization sounds helpful, and I want to try if it helps me have a better recovery.” – Interviewee 1, male, 70 years old*.

However, half of the participants had doubts. They assumed that too-early mobilization could result in negative consequences, such as screw failure or delayed bone healing.

*“Well, early mobilization sounds good, and I really would like to try it. However, I am concerned that it will result in screw failure.” – Interviewee 2, male, 68 years old*.

*“I am old, and bone heals slowly. Will it affect bone healing if I mobilize too early? Maybe I should lay down on my bed until the bone heals.” – Interviewee 15, female, 74 years old*.

In our study, 10 of the 24 participants interviewed completed early mobilization. Although they achieved successful mobilization with help from nurses in the early stage after surgery, they also expressed their concerns about the physical condition they were in and whether their safety was guaranteed. The use of monitoring devices seemed to increase their anxiety.

*“I have my blood pressure measured before and after mobilization. Do I have to be measured this frequently? This made me nervous.” – Interviewee 17, female, 65 years old*.

### Benefits

Following lumbar spinal surgery, there is significant variability in postoperative rehabilitation and advice offered by doctors, nurses, and physiotherapists. In the present study, we found that the patients urgently desired rapid recovery and relief from their preoperative condition, such as pain, numbness, and inconveniences (such as the inability to transfer themselves between the bed and chair, walk long distances, and climb stairs). Early mobilization after surgery is one choice for enhanced recovery, and the participants considered that early mobilization seemed to be a good sign of successful surgery and functional recovery.

*“I had been lying in bed for 2 months (before surgery). I hope I can recover as soon as possible. I accept the recommendation of early mobilization by my doctor. I am so missing my friends and my work.” – Interviewee 1, male, 70 years old*.

Although most of the participants expressed their wishes for early postoperative rehabilitation, half of them also acknowledged that they could not mobilize as early such as 4 h after surgery because they were uncertain about the functional results. They questioned the benefits of early mobilization and improvements of physical function that might be obtained.

*“To be honest, no matter how much you said about the advantages of early mobilization, I did not see that, and I do not know whether it is suitable for me.” –Interviewee 5, female, 62 years old*.

### Daily routines

Almost all participants described their concerns about the detailed movements necessary in their daily routines. They feared that incorrect performance of activities would offset the benefits gained from early mobilization and that being too active would result in negative physical consequences. These concerns often led them to be fearful of performing daily activities, attaining sleep comfort, returning to work, exercising, and preventing future disability.

*“I am a little upset about various situations in my daily routines.” – Interviewee 7, male, 69 years old*.

The most frequent question that participants raised was their sleeping posture. They complained that the sleeping posture advised by the nurse could not be maintained for a long period. They were also afraid of changing postures in bed, and the help provided by their families was thought to be unprofessional and sometimes led to worsening of pain or discomfort.

*“I do not know even how to sleep! I have to think if this posture is right for me.”– Interviewee 3, female, 71 years old*.

Another daily routine they were concerned about was how to manage themselves at home, such as how to get up from bed, when to walk, and how to perform exercise. In China, most patients prefer to go home rather than to a rehabilitation centre or a skilled nursing facility after discharge. In this study, all the participants were afraid of performing daily activities without help from healthcare professionals.

*“I still need assistance now, and I am trying hard to learn how to get up and walk correctly.” – Interviewee 15, female, 74 years old*.

The participants also asked about the recommended diet. They wondered what they should or should not eat or drink in their current physical condition, and they inquired about the most suitable diet for mobilization that would accelerate the recovery process.

### Pain

Pain is one of the most critical factors affecting early mobilization. The participants in the present study shared multiple stories about their surgical experiences and how they suffered from postoperative pain. They expressed a need for a postoperative recovery plan from their healthcare team to manage the pain, which could be severe at times. However, early mobilization is also a critical factor that introduces or increases postoperative pain.

*“I was seriously suffering from low back pain, and I wanted to solve this problem through surgery. However, I did not expect that that the pain would be worse after than before the operation. Even my legs became painful, and I could not walk right after surgery.”— Interviewee 16, female, 76 years old*.

*“I have severe low back pain since undergoing surgery. When I lie down, the symptom gets better. When I walk, the symptom gets worse. I do not want to walk.” – Interviewee 3, female, 71 years old*.

We asked the participants about the pain management strategies they used to effectively manage pain during mobilization. Patient-controlled analgesia (PCA) was the most commonly used pain relief method. Prescription opioid medications were also added to existing regimens for patients managing acute pain after mobilization. Postoperative pain made early mobilization even more difficult to perform. Although the participants understood that their pain might worsen during and after mobilization, they did not expect how severe the pain would be, what situations were acceptable, and when they should request a medical intervention. Moreover, sometimes they refused to mobilize rather than using a medical intervention to manage the increasing pain after mobilization.

*“I do not know at what level of pain I should take the medications and how they worked and how often should I take it.” – Interviewee 3, female, 71 years old*.

Some participants experienced adverse effects after using PCA or taking opioid medications. One participant said she was afraid of vomiting after using PCA, and she therefore endured the pain without using any medications. Some participants expressed a dislike for taking pain medications or an intention to use as few medications as possible, and they eventually stopped taking their prescriptions.

*“I do not want to rely on pain medications any longer. I have had enough.” – Interviewee 2, male, 68 years old*.

*“I will never take pain medications. Pain-relieving drugs are addictive. When I feel pain, I would prefer to suffer from it.”– Interviewee 16, male, 64 years old*.

By contrast, a few of the other participants took excessive pain medications when severe pain occurred after mobilization. They thought the pain should be relieved soon after taking the medications. If the symptom remained, they tended to continue to take more medication until it was effective.

*“The medication is not effective. I have to use it beyond the recommendations.” – Interviewee 8, female, 69 years old*.

### Education

Education is an essential part of increasing patients’ understanding and compliance with early mobilization after spinal surgery. Based on the preoperative appointment with the chief surgeon the day before surgery, the patients obtained information about the surgical plan and what to expect during and after surgery. Additionally, they were able to learn about early postoperative mobilization programs in their recovery process. At the time of our study, nurses mainly conducted the patients’ early mobilization education in our centre. The mobilization instructions were given to the patients in oral or written format, usually before the first-time mobilization after surgery. However, some participants complained that the information and instructions were too complicated and that they could not be easily and fully understood.

*“The doctor had a conversation with my family and me. He talked in great detail and very professionally about the surgery. However, I couldn’t understand his explanation. The doctor looked so busy. I was too embarrassed to ask any question, and I did not understand.” – Interviewee 8, female, 69 years old*.

Other participants also complained that the healthcare providers did not provide enough information about the significance of early mobilization and the severity of complications that can be caused by a prolonged period of lying in bed. They did not fully understand the concept and significance of early mobilization and were not aware enough of the potential complications that could occur if they were lying in bed for too long.

*“The nurses said something about it (complications of lying in bed), but I do not believe it will happen to me.” – Interviewee 6, male, 64 years old*.

Another concern raised by the participants was the different opinions on the timing of first-time mobilization among the surgeons, anaesthesiologists, physiatrists, and nurses. Moreover, even the chief nurses’ instructions sometimes differed from the chief surgeons’ instructions. It was difficult to correct the timing of first-time mobilization when it differed among surgeons, anaesthesiologists, physiatrists, or nurses, and such situations were likely to cause distrust, confusion, irritability, or antipathy in the patients.

*“The nurse told me that I could get out of bed 4 hours after surgery; however, the anaesthesiologist told me that I had to stay in bed for at least 12 hours.” – Interviewee 7, male, 69 years old*.

*“They do not have the same opinion on when should I get out of bed; they seem to have some disagreements. I feel it is difficult to trust any of them, even if they are my healthcare providers.” – Interviewee 12, male, 63 years old*.

A small portion of the participants also complained that what the healthcare providers told them was not the same as what they found on the internet. Some of them expressed their wishes to trust the information they obtained from the internet and the experience of other patients rather than the information they obtained from the doctors and nurses. One participant also stated that she believed in the advice obtained from “experts” on the internet through a paid consultation online.

*“One of my friends had the same surgery last year. He told me not to get up until 1 month after surgery. And I think he is right.” – Interviewee 10, male, 62 years old*.

### Support

One of the most important themes identified from the interview data was the need for family support. All the participants in our study chose to go home when discharged. Unlike patients in rehabilitation centres who can receive mobilization assistance from nurses, doctors, and rehabilitation therapists, patients at home can only rely on family members. However, according to the information we extracted from the interview data, lack of support was one of the most important factors hindering the patients’ enthusiasm for mobilization.

*“My husband has to go to work every day. It’s hard to mobilize all by myself. I can only get up for some activities after he comes back.” – Interviewee 9, female, 60 years old*.

Meanwhile, how the patient’s family members think about mobilization will also influence the patients by encouraging or discouraging them. If family members decline to provide help, the patients’ activities will decrease.

*“My wife thought it was not good for me to mobilize too much. She said when I discharge to home, I should do what she told me because she is my caregiver, not the nurse.” – Interviewee 2, male, 68 years old*.

In addition to receiving help, patients rely on their family members to know what to do and to problem-solve in the process of mobilization. Having suggestions for what to do when performing activities, as well as in a specific situations such as managing pain or dealing with tiredness, would be helpful for patients and families so that they know how to respond.

*“When I was mobilizing, she (daughter) had no idea about what she could do to encourage me.” – Interviewee 11, female, 64 years old*.

Other patients also expressed the hope that they would go to a rehabilitation centre or skilled nursing facility for further treatment after discharge. Although their family members were able to meet their needs and provide adequate help, they still tended to require the support of professionals. However, there were too few such rehabilitation centres to choose from, and the costs were high.

## Discussion

Immobilization after spinal surgery can be accompanied by several problems, such as urinary tract infection, hypostatic pneumonia, muscle weakness, skin pressure injury, and venous thromboembolism [4]. Recent studies have proven that early mobilization after spinal surgery can improve patients’ sense of well-being and long-term outcomes, and increasing healthcare providers’ recognition of its importance and benefits will encourage them to introduce it into clinical practice. However, there are still obstacles to the implementation of early mobilization. In the current study, the participants showed a low acceptance rate for early mobilization. Given their different education levels and cultural backgrounds, the participants expressed different understandings of early mobilization. Seven of 21 participants with an education level below a bachelor’s degree felt confused about the concept of early mobilization and doubted its necessity. The participants tended to selectively neglect the potential complications due to prolonged lying in bed and simply believed that such complications would never happen to them. Six of these seven participants assumed that ambulating too early or becoming too active might lead to other negative physical consequences, and they felt they should only resume activities after feeling fully recovered. However, it was difficult to achieve full recovery in a short period, and this often led to prolonged immobilization in bed for days and even weeks [21, 22].

With regard to patient care after lumbar spinal surgery, current guidelines advise early mobilization. However, the optimal timing for the first-time mobilization remains imprecise. In a retrospective study of patients who had undergone lumbar spinal fusion surgery, the patients were recommended to roll and walk to the doorway immediately after surgery [23]. Nonetheless, in the early mobilization protocol conducted by the Cleveland Clinic Foundation, all postoperative spinal surgery patients without contraindications were suggested to mobilize within 8 h after arriving at the regular nursing floor [24]. Confusion about the optimal time to initiate mobilization tends to make patients act more conservatively with reduced enthusiasm for early mobilization, often resulting in prolonged bed rest. In a retrospective study conducted by the Michigan Spine Surgery Improvement Collaborative, > 40% of 23,295 patients undergoing lumbar decompression and fusion surgery did not mobilize on the first postoperative day [25].

Disagreement regarding the timing of mobilization among healthcare providers will also lead to inconsistencies in patient education. Eight of the 24 participants in the current study complained that they had been provided inconsistent information by surgeons, anaesthesiologists, physiatrists, or nurses with regard to mobilization. Inconsistency will lead to distrust and antipathy among patients, and trust is the key to effective patient–physician relationships [26, 27]. When patients cannot obtain genuine care from their healthcare providers, good patient–provider relationships cannot be established. The patients’ trust in surgeons, anaesthesiologists, physiatrists, and nurses will be difficult to recover, lowering patients’ compliance with postoperative mobilization.

Despite evidence-based research recommending that patients initiate mobilization early after surgery, there are still no detailed guidelines for the optimal time and procedures to initiate mobilization. Unequivocal guidance issued by a professional committee or government is urgently needed for clinical practice. Furthermore, the hospital itself should unify the procedures and regulations of postoperative early mobilization to enhance patient education by healthcare providers. Patient education provided by hospitals, surgeons, and nurses is an essential part of postoperative mobilization. A multidisciplinary mobilization protocol should be issued by administrative staff, spine surgeons, nurses, rehabilitation therapists, anaesthetists, and nutritionists after approval by the faculty committee and ethics committee. Spine surgeons, nurses, and rehabilitation therapists should be thoroughly familiar with the multidisciplinary protocol, and the protocol should be posted at nurses’ stations and in restrooms to improve remembrance. One-to-one education should be provided if conditions allow. In addition to the content, the timing of education is also important for improving patient compliance with a multidisciplinary mobilization protocol. Patient education is better initiated from the first-time appointment with surgeon preadmission [28]. Education is continuous and should be carried out before, during, and after surgery. In addition to oral and written education forms, video education and a mobile app that can provide more effective preoperative education should also be considered. It is essential to ensure that the patient fully understands the mobilization protocol, the functional aspect of the physical activities that he or she will be performing, and the management of analgesics at home [23].

Among the participants who agreed with the concept of early postoperative mobilization in the present study, the most frequently mentioned concern regarding mobilization was pain. Ten of 17 participants considered postoperative pain to be the main reason for their refusal to mobilize, and this result was consistent with previous studies [29, 30]. Lumbar decompression and fusion surgery is among the six most painful procedures [31], and patients often experience a high level of pain on the first postoperative day. Pain has detrimental effects on patients’ enthusiasm for postoperative mobilization. Some participants expressed concerns about the increasing pain induced by mobilization, which had become a major hindrance to their early mobilization program. Postoperative pain management is challenging, and procedure-specific pain management plans are needed. A comprehensive pain management plan should be designed in partnership with the patient, spine surgeons, nurses, and the patient’s family caregivers [29]. This pain plan should be part of the multidisciplinary mobilization protocol. Patients should be informed about the need for both nonopioid medications and nonpharmacologic strategies [29]. Some studies have shown a consistent association between opioid use and worse outcomes after spinal surgery [32, 33]. By contrast, an opioid-free model showed no significant differences in pain control or postoperative opioid consumption [34]. An adequate pain management plan for postoperative mobilization should be comprehensive and offer not only pharmacological strategies but also physical modalities. This will give patients the best opportunity to find a successful combination of strategies to control pain during mobilization and increase their physical activities.

Other participants (3 of 17) also complained that the monitoring devices used to identify adverse effects decreased their enthusiasm for mobilization. The monitoring devices recorded the patients’ vital signs after surgery; however, staying connected to these machines restricted the patients’ mobility, making them unable to leave the bed. When monitoring devices accompany the patients during mobilization, the patients may feel that this is cumbersome, uncomfortable, and upsetting, and their enthusiasm for mobilization will decrease. Although wearable and wireless monitors could offer a solution, these medical devices remain unavailable in the current stage of clinical practice [7].

The results of the current study illustrate the importance of family support for the patient in the postoperative recovery process. Of the 24 patients in our study, 18 shared the attitude that support from their family members was essential to their continuance of mobilization. For better patient care, however, family members must know how to assist with mobilization, what may happen, what limitations the patient will face during mobilization, and what the patient will need after mobilization. This information will enable family members to make realistic plans to best assist the patient in their mobilization. If possible, patients should be encouraged to bring their family members to the preoperative appointment so that they may be educated together about the multidisciplinary mobilization protocol, including a pain management plan. Patients’ family members may benefit from addressing preparatory information and coping skills while receiving education from healthcare providers [35].

### Limitations

In this qualitative study involving semi-structured interviews, we were able to achieve a deeper understanding of patients’ experiences and concerns about early mobilization. However, the study still had several limitations. First, the data collection was cross-sectional in nature rather than longitudinal, and the results were limited to one academic medical centre. Therefore, the data may not be representative of other settings. Second, like other exploratory studies, this study was designed for a specific category of patients, which may limit its generalizability. Third, all patients were relatively positive and willing to express their feelings; therefore, to some extent, our data do not fully demonstrate the factors that hinder early mobilization, and our results might lack the experience and concerns of patients who are unwilling to share. In addition, we did not interview staff members. Notably, their concerns and views on early postoperative mobilization are equally as important as patients’. In future research, we may explore more ideas from patients unwilling to mobilize early as well as from staff to identify the underlying factors obstructing the application of early mobilization.

## Conclusions

This study revealed six themes of elderly patients’ experiences and concerns regarding early mobilization following lumbar spinal surgery. The findings can be used to guide future interventions for postoperative early mobilization in elderly patients. Clear guidance regarding the early mobilization and multidisciplinary mobilization protocol, including a comprehensive pain management plan, is essential for ensuring effective patient education by healthcare providers. This may have a positive effect on reducing patients’ stress and anxiety regarding postoperative early mobilization. Furthermore, education is intended solely for the patient but also extends to family members and all support caregivers who play a role in the successful implementation of early mobilization.

## Data Availability

All data generated or analyzed during this study are included in this published article.
